# Metabolic Reprogramming Driven by Modifiable Lifestyle Factors in Metabolic Syndrome and Alcohol-Related Liver Disease: A Narrative Review

**DOI:** 10.3390/metabo16040224

**Published:** 2026-03-30

**Authors:** Daniela Mihăilă, Horațiu-Paul Domnariu, Doru-Florian-Cornel Moga, Carmen-Daniela Domnariu

**Affiliations:** 1 Doctoral School of Medicine, Faculty of Medicine, “Lucian Blaga” University of Sibiu, 550024 Sibiu, Romania; 2 Department of Internal Medicine, “Dr. Alexandru Augustin” Military Clinical Emergency Hospital Sibiu, 550024 Sibiu, Romania; 3 Surgical Clinical Department, Faculty of Medicine, “Lucian Blaga” University of Sibiu, 550024 Sibiu, Romania; horatiupaul.domnariu@ulbsibiu.ro (H.-P.D.); cornel.moga@ulbsibiu.ro (D.-F.-C.M.); 4 Clinical Department of Surgery, “Dr. Alexandru Augustin” Military Clinical Emergency Hospital Sibiu, 550024 Sibiu, Romania; 5 Department of Dental Medicine and Nursing, Faculty of Medicine, “Lucian Blaga” University of Sibiu, 550024 Sibiu, Romania; carmen.domnariu@ulbsibiu.ro

**Keywords:** metabolic syndrome, alcohol-related liver disease, metabolic reprogramming, lifestyle factors, insulin resistance, oxidative stress, mitochondrial dysfunction, MetALD

## Abstract

**Highlights:**

**What are the main findings?**

- Harmful lifestyle exposures (alcohol, smoking, unhealthy diet, physical inactivity) converge on shared metabolic reprogramming pathways—insulin resistance, dysregulated lipid metabolism, mitochondrial dysfunction, oxidative stress, and chronic low-grade inflammation—linking metabolic syndrome with alcohol-related liver injury.
- The coexistence of metabolic dysfunction and alcohol exposure is consistently associated with synergistic worsening of liver outcomes, supporting the emerging metabolic alcohol-related liver disease (MetALD) phenotype within a metabolic–hepatic continuum.

**What are the implications of the main findings?**

- Bidirectional clinical screening is warranted: assess metabolic syndrome components in individuals with harmful alcohol use and evaluate alcohol consumption patterns in patients with metabolic dysfunction to identify high-risk MetALD profiles earlier.
- Integrated lifestyle-based management (alcohol reduction/abstinence support, smoking cessation, diet quality improvement, and physical activity promotion), alongside cardiometabolic risk optimization, may provide synergistic benefits for preventing and mitigating MetALD progression.

**Abstract:**

Background: Metabolic syndrome (MetS) and alcohol-related liver disease (ALD) are increasingly recognized as interconnected disorders linked by shared mechanisms of lifestyle-driven metabolic reprogramming. Alterations in systemic and hepatic metabolic pathways—including insulin signaling, lipid metabolism, mitochondrial bioenergetics, and redox homeostasis—reduce hepatic resilience to alcohol exposure and accelerate liver disease progression. Objective: This narrative review aims to integrate clinical, epidemiological, and mechanistic evidence published over the past two decades to examine how modifiable lifestyle factors contribute to metabolic reprogramming linking metabolic syndrome and alcohol-related liver disease with prioritization of high-level clinical evidence (cohort studies, meta-analyses, and guidelines). Key Findings: Modifiable lifestyle exposures such as alcohol consumption, cigarette smoking, unhealthy dietary patterns, and physical inactivity converge on common metabolically mediated pathways, including insulin resistance, dysregulated lipid metabolism and lipotoxicity, mitochondrial dysfunction, oxidative stress, chronic low-grade inflammation, and gut–liver axis perturbations. These processes are reflected in altered metabolite profiles involving lipid species, bile acids, tricarboxylic acid cycle intermediates, and microbiota-derived metabolites, shaping a metabolic–hepatic continuum. Among these, alcohol consumption and metabolic dysfunction show the strongest and most consistent associations with liver disease progression, with evidence supporting synergistic rather than additive effects. Conclusions: The coexistence of metabolic dysfunction and alcohol exposure is consistently associated with synergistic worsening of liver-related outcomes, including fibrosis progression, cirrhosis, and hepatocellular carcinoma. Recognition of metabolic alcohol-related liver disease (MetALD) underscores the need for integrated lifestyle-based strategies targeting alcohol consumption, smoking cessation, dietary quality, and physical activity to modulate shared metabolic and inflammatory pathways. A metabolically informed, systems-level approach may improve risk stratification, prevention, and management across the metabolic–hepatic continuum.

## 1. Introduction

Metabolic syndrome (MetS) and alcohol-related liver disease (ALD) are increasingly conceptualized as interconnected conditions driven by lifestyle-induced metabolic reprogramming. This process affects systemic and hepatic metabolic pathways governing glucose and lipid fluxes, mitochondrial bioenergetics, and redox homeostasis. Perturbations in metabolite profiles—including alterations in lipidomic signatures, tricarboxylic acid cycle intermediates, bile acids, and oxidative stress–related metabolites—represent a mechanistic interface linking lifestyle exposures to insulin resistance and inflammation. These alterations contribute to progressive liver injury and reduced hepatic resilience to alcohol exposure.

Metabolic reprogramming refers to adaptive alterations in cellular and systemic metabolic pathways that occur in response to environmental, nutritional, and inflammatory stimuli. These changes involve coordinated shifts in glucose utilization, lipid metabolism, mitochondrial bioenergetics, and redox balance, allowing cells and tissues to adjust energy production and biosynthetic processes to altered physiological demands. In metabolic disorders and chronic liver diseases, metabolic reprogramming becomes maladaptive, promoting insulin resistance, lipotoxicity, oxidative stress, and inflammatory signaling that collectively contribute to hepatic injury and disease progression.

At the molecular level, metabolic reprogramming is orchestrated through several interconnected signaling pathways. Key regulatory nodes include AMP-activated protein kinase (AMPK), mechanistic target of rapamycin (mTOR), and peroxisome proliferator-activated receptors (PPARs), which coordinate nutrient sensing, lipid oxidation, and mitochondrial function. Dysregulation of these pathways alters hepatic glucose and lipid fluxes, promotes accumulation of free fatty acids, and impairs mitochondrial β-oxidation. These processes enhance reactive oxygen species generation and activate inflammatory cascades involving NF-κB and other stress-response pathways, thereby linking metabolic dysfunction with progressive liver injury.

In the context of metabolic syndrome and alcohol-related liver disease, metabolic reprogramming represents a critical mechanistic interface through which lifestyle exposures influence disease susceptibility. Excess caloric intake, alcohol consumption, smoking, and physical inactivity induce coordinated metabolic shifts affecting insulin signaling, lipid handling, and mitochondrial bioenergetics. These alterations reduce hepatic metabolic flexibility and increase vulnerability to alcohol-induced oxidative and inflammatory injury, thereby accelerating disease progression along a metabolic–hepatic continuum.

Metabolic syndrome (MetS) is characterized by a constellation of interrelated cardiometabolic abnormalities, including central obesity, insulin resistance, dyslipidemia, and arterial hypertension, which collectively increase the risk of type 2 diabetes mellitus, cardiovascular disease, and liver pathology [1,2,3]. In parallel, alcohol-related liver disease (ALD) remains a leading cause of chronic liver injury worldwide, encompassing a disease spectrum ranging from hepatic steatosis to cirrhosis and hepatocellular carcinoma (HCC) [4,5].

Recent updates in steatotic liver disease nomenclature have introduced the term metabolic dysfunction-associated steatotic liver disease (MASLD) to replace the former NAFLD terminology. MASLD encompasses a spectrum of liver disorders associated with metabolic dysfunction, including metabolic dysfunction–associated steatohepatitis (MASH). In parallel, alcohol-related liver disease (ALD), also referred to as alcohol-associated liver disease (ArLD), describes liver injury primarily driven by harmful alcohol consumption. Within the updated taxonomy, metabolic alcohol-related liver disease (MetALD) has been proposed to describe individuals presenting with both metabolic dysfunction and clinically relevant alcohol exposure. Although metabolic syndrome (MetS) represents only one manifestation of metabolic dysfunction, it provides a clinically recognizable cluster of cardiometabolic risk factors frequently overlapping with the MASLD spectrum. Clarifying these definitions helps contextualize the metabolic–hepatic continuum explored in this review. To clarify the conceptual relationships among these entities, a comparative overview of MetS, ALD, MASLD, and MetALD is presented in Table 1.

Beyond excessive alcohol consumption, accumulating evidence indicates that metabolic dysfunction critically modulates susceptibility to alcohol-induced liver injury. Obesity, insulin resistance, and dyslipidemia amplify ethanol-related hepatotoxicity by promoting oxidative stress, mitochondrial dysfunction, chronic inflammation, and hepatic fibrogenesis [6,7,8,9]. Consequently, MetS and ALD frequently coexist, forming a metabolic–hepatic continuum characterized by accelerated disease progression and unfavorable clinical outcomes [10,11,12,13].

Lifestyle behaviors, including alcohol consumption patterns, cigarette smoking, dietary habits, and physical activity, are key determinants of metabolic homeostasis. These modifiable factors influence insulin sensitivity, lipid metabolism, inflammatory signaling, and hepatic fat accumulation—processes central to the pathophysiology of both MetS and ALD [14,15,16,17,18,19,20,21,22,23,24,25,26]. As such, they represent important targets for preventive and therapeutic strategies [9,27,28,29].

Given the shared metabolic basis of these conditions and the growing recognition of their interaction, a comprehensive synthesis of available evidence is warranted. This narrative review aims to evaluate the impact of modifiable lifestyle risk factors on MetS and ALD, with particular emphasis on metabolically mediated mechanisms underlying disease susceptibility and progression [21,26,30].

Sex-related differences may further modulate the relationship between metabolic syndrome and alcohol-related liver disease. Clinical and epidemiological evidence indicates that women develop alcohol-related liver injury at lower cumulative alcohol exposure and shorter drinking duration compared with men. This increased susceptibility has been attributed in part to lower gastric alcohol dehydrogenase activity, higher blood alcohol concentrations for equivalent alcohol intake, and differences in body composition and total body water.

At the mechanistic level, sex hormones also influence hepatic lipid metabolism, inflammatory signaling, and mitochondrial function. Estrogen signaling has been shown to modulate oxidative stress responses and immune activation, potentially enhancing inflammatory and fibrogenic pathways in the context of chronic alcohol exposure. In addition, sex-related differences in insulin sensitivity, adipose tissue distribution, and lipid handling may influence susceptibility to lifestyle-induced metabolic reprogramming and disease progression.

These observations highlight biological sex as an important modifier of metabolic and hepatic risk and support the need for sex-disaggregated analyses in future studies investigating the interaction between metabolic dysfunction and alcohol exposure [31].

## 2. Literature Overview

This narrative review is based on an integrative appraisal of clinical, epidemiological, and mechanistic literature addressing the interplay between metabolic syndrome and alcohol-related liver disease. The evidence base predominantly includes observational studies, systematic reviews, meta-analyses, and relevant clinical guidelines published between 2000 and 2025, with particular emphasis on modifiable lifestyle factors such as alcohol consumption, smoking, diet, and physical activity. The literature search was conducted using major biomedical databases, including PubMed/MEDLINE, Scopus, and Web of Science, to identify relevant studies published between January 2000 and December 2025. Search terms included combinations of keywords such as “metabolic syndrome”, “alcohol-related liver disease”, “alcohol-associated liver disease”, “MetALD”, “metabolic reprogramming”, “lifestyle factors”, “insulin resistance”, “oxidative stress”, “diet”, “physical activity”, and “smoking”. Reference lists of relevant articles and reviews were also screened to identify additional studies. Studies focusing on viral hepatitis or other primary chronic liver diseases unrelated to alcohol exposure were excluded to maintain the conceptual focus of the review. Priority was given to human studies, systematic reviews, meta-analyses, cohort studies, and clinical guidelines.

Rather than applying a formal systematic review methodology, the literature was synthesized conceptually to contextualize population-level associations within established and emerging mechanisms of lifestyle-driven metabolic reprogramming. Key themes were identified across studies, including insulin resistance, dysregulated lipid metabolism, mitochondrial dysfunction, oxidative stress, chronic low-grade inflammation, and gut–liver axis alterations, which together underpin the metabolic–hepatic continuum linking metabolic syndrome and alcohol-related liver disease.

Throughout this review, the terms metabolic syndrome (MetS), alcohol-related liver disease (ALD), and metabolic alcohol-related liver disease (MetALD) are used consistently in accordance with established clinical and pathophysiological definitions.

In synthesizing the available evidence, priority was given to higher levels of clinical evidence, including longitudinal cohort studies, randomized controlled trials, meta-analyses, and international clinical practice guidelines. Observational studies were interpreted in the context of consistency across populations and study designs. When heterogeneous or conflicting findings were reported, greater weight was attributed to large population-based analyses and synthesis-level evidence. Mechanistic and experimental studies were incorporated primarily to support biological plausibility and to contextualize clinical observations rather than to replace clinical inference derived from human studies. This integrative approach allowed epidemiological associations to be interpreted within a mechanistic framework while maintaining a clinically oriented perspective.

## 3. Lifestyle Factors as Drivers of Metabolic Reprogramming

### 3.1. Alcohol Consumption and Systemic Metabolism

Multiple cohort studies and meta-analyses demonstrated a dose-dependent association between alcohol consumption and the risk of developing metabolic syndrome (MetS) [32,33,34,35]. Longitudinal analyses indicated that increases in alcohol intake over time were associated with a higher incidence of MetS, whereas reductions in consumption attenuated metabolic risk [32,33]. Alcohol exposure was consistently linked to insulin resistance, dyslipidemia, and adverse metabolic profiles, supporting its role as a modifiable determinant of metabolic dysfunction.

The relationship between alcohol consumption and metabolic syndrome risk is also influenced by drinking patterns, beverage type, and biological sex. Evidence suggests that heavy episodic or binge drinking is associated with a substantially higher risk of metabolic disturbances compared with moderate, regular consumption patterns. In contrast, observational studies have reported heterogeneous associations between specific alcoholic beverages and metabolic outcomes, potentially reflecting differences in accompanying dietary patterns and lifestyle behaviors rather than intrinsic beverage-specific effects.

Sex-specific differences further modulate these associations, as women generally exhibit higher blood alcohol concentrations for equivalent alcohol intake due to lower total body water and reduced first-pass metabolism. Consequently, metabolic and hepatic effects of alcohol exposure may occur at lower consumption thresholds in women compared with men, highlighting the importance of considering sex-specific vulnerability when evaluating alcohol-related metabolic risk.

At the population level, global burden analyses further identified alcohol consumption as a major contributor to cardiometabolic morbidity and mortality, reinforcing its relevance as a public health risk factor with systemic metabolic consequences [36,37].

Overall, alcohol consumption emerges as the most consistently and strongly associated lifestyle factor driving both metabolic dysfunction and liver disease progression. Evidence from cohort studies and meta-analyses demonstrates a robust, dose-dependent relationship between alcohol intake and adverse metabolic and hepatic outcomes, further modified by drinking patterns, with binge drinking conferring additional risk beyond average intake. Importantly, alcohol acts not only as an independent hepatotoxic agent but also as a key amplifier of metabolic vulnerability, lowering the threshold for liver injury in individuals with underlying metabolic dysfunction.

Compared with other lifestyle exposures, alcohol shows the strongest and most consistent association with clinically relevant outcomes, including fibrosis progression, cirrhosis, and hepatocellular carcinoma. However, uncertainty remains regarding safe consumption thresholds, particularly in metabolically at-risk populations, and the extent to which moderate intake may exert heterogeneous effects depending on sex, genetic background, and coexisting lifestyle factors.

### 3.2. Alcohol Consumption and Alcohol-Related Liver Disease

Across observational studies and synthesis-level evidence, excessive alcohol consumption emerged as the primary determinant of alcohol-related liver disease (ALD) development and progression [4,5,9]. Alcohol exposure was consistently associated with hepatic steatosis, progressive fibrosis, cirrhosis, and liver related mortality.

Evidence supporting dose and pattern dependent hepatotoxicity was reinforced by studies linking overall alcohol exposure and drinking patterns to liver outcomes and disease burden. In particular, binge drinking patterns were associated with an increased risk of liver related events, independent of average alcohol intake [30,38].

Beyond alcohol consumption, additional lifestyle exposures such as smoking, diet quality, and physical activity further modulate metabolic reprogramming and liver disease risk.

### 3.3. Interaction Between Metabolic Syndrome and ALD (MetALD)

Several studies evaluated the combined effects of metabolic dysfunction and alcohol consumption on liver outcomes, consistently demonstrating synergistic interactions [6,7,8,9,10,11,39,40,41]. Quantitative analyses from large population-based cohorts further support this interaction. For example, individuals with both metabolic syndrome and harmful alcohol consumption exhibit significantly higher risks of advanced liver disease compared with those with either exposure alone, with hazard ratios for severe liver outcomes generally ranging between two- and fourfold depending on the population and exposure thresholds examined. These studies also report accelerated fibrosis progression and increased risks of cirrhosis and hepatocellular carcinoma when metabolic dysfunction and alcohol exposure coexist, highlighting the clinically relevant magnitude of this interaction. More detailed clinical evidence further illustrates this effect. In the Finnish Health 2000 cohort, diabetes among alcohol risk users was associated with a substantially increased risk of severe liver outcomes (HR 6.79, 95% CI 3.18–14.5). Similarly, in a biopsy-controlled cohort of patients with alcohol-related liver disease, insulin resistance (HOMA-IR ≥ 2.5) independently predicted higher fibrosis stage (OR 3.04, 95% CI 1.90–4.87). These findings reinforce the concept that metabolic dysfunction amplifies liver-related risk in alcohol-exposed individuals.

Emerging conceptual frameworks and clinical updates have described this dual etiology interaction as metabolic alcohol-related liver disease (MetALD), emphasizing the role of metabolic dysfunction in amplifying alcohol-induced hepatotoxicity and shaping disease trajectories [13,42,43].

Overall, the clinical literature consistently identifies alcohol consumption as the dominant lifestyle determinant of liver-related outcomes, including fibrosis progression, cirrhosis, and hepatocellular carcinoma. Importantly, the presence of metabolic dysfunction appears to lower the threshold for alcohol-induced liver injury, suggesting that alcohol and metabolic risk factors interact synergistically rather than additively. While the dose–response relationship between alcohol intake and liver disease progression is well established, uncertainty persists regarding safe consumption thresholds in individuals with metabolic vulnerability.

### 3.4. Interactions Between Drugs, Alcohol, and Metabolic Dysfunction

Beyond the synergistic interaction between metabolic syndrome and alcohol consumption, concomitant drug exposure may further modulate metabolic reprogramming and liver disease progression. Patients with metabolic syndrome frequently receive multiple pharmacological therapies, including antidiabetic agents, lipid-lowering drugs, antihypertensive medications, and psychoactive compounds, many of which undergo hepatic metabolism and may interact with alcohol-related metabolic and inflammatory pathways [9,44]. Certain medications may exhibit clinically relevant hepatotoxic interactions when combined with alcohol exposure. For example, chronic alcohol consumption induces cytochrome P450 2E1 (CYP2E1), enhancing the formation of toxic metabolites from drugs such as acetaminophen and increasing the risk of hepatocellular injury. In addition, medications commonly used in patients with metabolic syndrome—including statins, antidiabetic agents, and certain psychoactive drugs—may further influence hepatic metabolic pathways and oxidative stress responses, potentially amplifying liver vulnerability in individuals with combined metabolic dysfunction and alcohol exposure.

Alcohol consumption can significantly influence drug metabolism through modulation of hepatic cytochrome P450 enzymes, particularly CYP2E1, thereby affecting drug bioavailability, oxidative stress generation, and hepatotoxic risk [8,9,44]. Chronic alcohol exposure induces CYP2E1 activity, which enhances the production of reactive oxygen species and may increase the formation of toxic drug metabolites, amplifying liver injury, especially in individuals with underlying metabolic dysfunction [8,44].

Conversely, metabolic abnormalities such as insulin resistance, obesity, and hepatic steatosis may alter drug pharmacokinetics and pharmacodynamics through changes in hepatic lipid accumulation, mitochondrial function, and inflammatory signaling [25,29]. These metabolic alterations can modify drug distribution and clearance, potentially exacerbating drug–alcohol interactions and cumulative hepatotoxicity.

In this context, polypharmacy, which is common among patients with metabolic syndrome, may represent an additional layer of complexity within the MetALD spectrum. The combined exposure to alcohol, metabolic dysfunction, and hepatically metabolized drugs may act synergistically to promote oxidative stress, chronic inflammation, and fibrogenesis, thereby accelerating disease progression [9,29,44]. These interactions underscore the importance of careful medication review and individualized risk assessment in patients with combined metabolic dysfunction and alcohol exposure.

Although studies focusing on viral hepatitis were excluded from the present synthesis, prior evidence from other chronic liver disease settings suggests that lipid-lowering therapies such as statins may exert pleiotropic metabolic and anti-inflammatory effects, with potential implications for hepatic outcomes [45].

### 3.5. Smoking

Cigarette smoking was consistently associated with an increased incidence and prevalence of metabolic syndrome (MetS) in a dose-dependent manner across cohort studies [14,15,16,17]. Smoking exposure was linked to insulin resistance and adverse metabolic profiles, contributing to systemic metabolic dysfunction.

In the context of alcohol-related liver disease (ALD), smoking appears to act as a disease modifier, contributing to liver disease progression through oxidative stress and pro-inflammatory mechanisms [8,9]. Smoking-related toxic compounds increase reactive oxygen species production, impair antioxidant defenses, and promote inflammatory signaling, thereby exacerbating hepatocellular injury.

Moreover, accumulating evidence suggests that cigarette smoking may contribute to accelerated liver fibrosis by promoting hepatic stellate cell activation and fibrogenic responses, particularly in individuals with concomitant alcohol exposure and metabolic dysfunction. These mechanisms may help explain the observed association between smoking and more advanced liver disease stages and fibrosis progression in patients with ALD [8,14].

Overall, cigarette smoking demonstrates a consistent but comparatively moderate association with metabolic dysfunction and liver disease progression. Cohort studies support a dose-dependent relationship between smoking and metabolic syndrome; however, its independent contribution to advanced liver outcomes remains less well quantified than that of alcohol. Mechanistically, smoking contributes to hepatic injury through oxidative stress, inflammatory signaling, and fibrogenic activation, particularly in the presence of concomitant alcohol exposure and metabolic dysfunction. However, much of the available evidence is observational, and disentangling the independent effects of smoking from coexisting lifestyle risk factors remains challenging. Compared with alcohol, smoking is best characterized as a disease modifier rather than a primary driver of liver disease progression, although its cumulative impact may be clinically meaningful in high-risk populations.

### 3.6. Physical Activity

Regular moderate to vigorous physical activity was consistently associated with a reduced risk of MetS and improved cardiometabolic profiles across population-based studies and meta-analyses [19,20].

Among individuals with harmful alcohol use, higher levels of physical activity were associated with a lower incidence of ALD, suggesting a potential protective effect even in high-risk populations [18]. Additional cohort data linked physical activity to reduced liver fibrosis risk, supporting its role as a beneficial modulator of metabolic and hepatic outcomes [26].

Across observational studies and meta-analyses, regular physical activity demonstrates consistent protective associations with metabolic syndrome and liver disease risk. Improvements in insulin sensitivity, mitochondrial function, and hepatic lipid metabolism represent plausible mechanisms underlying these benefits. However, most available data remain observational, and optimal exercise thresholds for reducing alcohol-related liver disease risk in metabolically vulnerable populations remain incompletely defined.

### 3.7. Diet and Nutritional Factors

Western dietary patterns have been consistently associated with metabolic deterioration, insulin resistance, and an increased risk of metabolic syndrome (MetS), reflecting high intakes of saturated fats, refined carbohydrates, and ultra-processed foods [21]. In nutritional epidemiology, the Western dietary pattern is typically characterized by high consumption of energy-dense and ultra-processed foods, including red and processed meats, refined grains, sugar-sweetened beverages, saturated fats, and added sugars, often accompanied by low intake of fruits, vegetables, whole grains, and dietary fiber. These patterns are frequently operationalized in cohort studies using dietary pattern scores or indices derived from food frequency questionnaires. In contrast, adherence to Mediterranean-style dietary patterns, characterized by higher consumption of fruits, vegetables, whole grains, and unsaturated fats, was associated with improved metabolic outcomes and favorable cardiometabolic profiles across diverse populations [46]. However, the magnitude and consistency of these benefits may vary across populations depending on regional dietary habits, cultural contexts, genetic background, and differences in baseline metabolic risk. Moreover, much of the available evidence derives from observational studies conducted predominantly in Mediterranean and European populations, which may limit the generalizability of these findings to other geographic regions and dietary environments.

Beyond these widely studied dietary models, regional variations in dietary patterns may also influence metabolic and hepatic outcomes. Nutritional factors and overall metabolic control have been shown to modulate alcoholic fatty liver risk and disease progression, underscoring the importance of diet quality in alcohol-exposed populations.

Recent synthesis-level evidence further highlights the interaction between dietary quality and alcohol exposure in shaping liver risk trajectories, suggesting that unhealthy dietary patterns may exacerbate alcohol-related liver injury, whereas higher-quality diets may partially mitigate metabolic and hepatic risk [9,23,47]. Collectively, these findings emphasize that dietary patterns play a central role in metabolic reprogramming and ALD progression, with potential relevance across different cultural and regional contexts.

Overall, dietary patterns represent an important but more heterogeneous determinant of metabolic and hepatic risk compared with alcohol consumption. Western dietary patterns are consistently associated with adverse metabolic profiles and increased risk of metabolic syndrome, whereas Mediterranean-style diets are linked to improved cardiometabolic outcomes. However, the magnitude and consistency of these associations vary across populations and are influenced by regional dietary habits, cultural context, genetic background, and baseline metabolic risk. In contrast to alcohol, the effects of diet on liver disease progression are less directly quantified and appear to be mediated primarily through modulation of metabolic pathways rather than direct hepatotoxic mechanisms. Much of the available evidence is observational and context-dependent, limiting causal inference and generalizability. Importantly, diet interacts with other lifestyle exposures, particularly alcohol consumption, suggesting that its impact on liver disease risk is best understood within a broader behavioral and metabolic context rather than as an isolated determinant.

### 3.8. The Gut–Liver Axis and Lifestyle-Induced Metabolic Reprogramming

The gut–liver axis represents a critical interface linking lifestyle factors, metabolic dysfunction, and liver disease progression. Substantial evidence indicates that alterations in gut microbiota composition and intestinal barrier integrity contribute to metabolic reprogramming and hepatic injury by facilitating the translocation of microbial-derived products, particularly lipopolysaccharides, from the gut to the liver via the portal circulation [48,49]. This process promotes hepatic inflammatory signaling, oxidative stress, and insulin resistance, thereby exacerbating liver damage.

Both alcohol consumption and metabolic syndrome independently disrupt gut homeostasis. Chronic alcohol exposure has been shown to induce gut dysbiosis and increase intestinal permeability through impairment of tight junction proteins, while metabolic dysfunction is associated with altered microbial composition, low-grade systemic inflammation, and dysregulated bile acid signaling [49,50]. When combined, these factors may synergistically amplify gut-derived inflammatory signaling and contribute to lifestyle-induced metabolic reprogramming.

Emerging evidence further suggests that gut-derived metabolites, including short-chain fatty acids and bile acid derivatives, influence hepatic lipid metabolism, mitochondrial function, and insulin sensitivity, thereby shaping the metabolic–hepatic continuum and susceptibility to alcohol-related liver disease [50,51].

Although the precise contribution of gut microbiota alterations to metabolic alcohol-related liver disease (MetALD) remains incompletely defined, consideration of the gut–liver axis provides an integrative framework linking diet quality, alcohol exposure, metabolic dysfunction, and hepatic injury. Several gut microbial taxa have been implicated in bile acid metabolism and ethanol-related metabolic pathways. For example, genera such as Bacteroides, Clostridium, Lactobacillus, and Bifidobacterium contribute to bile acid biotransformation through bile salt hydrolase activity and the conversion of primary to secondary bile acids. In addition, certain intestinal bacteria including Escherichia coli, Klebsiella pneumoniae, and Enterococcus species have been associated with ethanol metabolism and endogenous ethanol production, which may further contribute to hepatic inflammation and metabolic dysregulation through the gut–liver axis.

At the molecular level, gut-derived lipopolysaccharide (LPS) is recognized by the LPS-binding protein (LBP)–CD14–MD-2 complex, which facilitates activation of Toll-like receptor 4 (TLR4) on Kupffer cells and other hepatic cell types. TLR4 signaling then proceeds through two major downstream pathways. The MyD88-dependent branch activates IRAK and TRAF6, promoting NF-κB and MAPK signaling and increasing the transcription of pro-inflammatory mediators such as tumor necrosis factor-α, interleukin-6, and interleukin-1β. In parallel, TLR4 internalization can initiate TRIF-dependent signaling, leading to TBK1/IKKε-mediated activation of IRF3 and interferon-related inflammatory responses. Recent research further suggests that these pathways interact with NLRP3 inflammasome activation and pyroptotic signaling, thereby amplifying hepatocellular injury, fibrogenic responses, and metabolic dysregulation along the gut–liver axis. In alcohol-related and metabolically mediated liver injury, sustained LPS exposure may therefore act not only as a trigger of inflammatory cytokine production but also as an upstream driver of oxidative stress, innate immune activation, and mitochondrial dysfunction [52].

## 4. Metabolic Dysfunction and Alcohol-Related Liver Disease

### 4.1. The MetALD Framework

Multiple cohort studies demonstrate that the coexistence of metabolic dysfunction and alcohol consumption is associated with accelerated fibrosis progression, increased cirrhosis risk, and higher incidence of hepatocellular carcinoma compared with either condition alone [51,53,54,55]. This synergistic interaction underpins the MetALD framework, which integrates metabolic and alcohol-related drivers of liver disease within a unified pathogenic model [13].

### 4.2. Mitochondrial Dysfunction, Oxidative Stress, and Inflammation

Both metabolic syndrome and chronic alcohol exposure disrupt mitochondrial β-oxidation and respiratory chain efficiency, leading to excessive reactive oxygen species production and oxidative stress [56,57]. Chronic low-grade inflammation further reinforces this pathogenic synergy, with adipose tissue-derived cytokines and alcohol-induced innate immune activation converging to promote hepatic fibrogenesis and disease progression [58,59]. Experimental evidence further implicates inflammatory and profibrogenic pathways such as NF-κB and transforming growth factor-β (TGF-β) signaling in mediating hepatocellular injury and fibrogenesis associated with metabolic and alcohol-related liver disease [60].

At the mitochondrial level, alcohol exposure and metabolic dysfunction are associated with impaired β-oxidation of fatty acids and reduced oxidative phosphorylation efficiency. Experimental and clinical studies indicate that chronic alcohol exposure disrupts mitochondrial respiratory chain activity, leading to decreased ATP production and accumulation of lipid intermediates within hepatocytes. These alterations are accompanied by increased generation of reactive oxygen species and mitochondrial DNA damage, which further aggravate oxidative stress and hepatocellular injury. Collectively, impaired fatty acid oxidation and reduced mitochondrial energy production represent key metabolic features linking lifestyle-induced metabolic reprogramming to progressive liver injury.

### 4.3. The Gut–Liver Axis

Alcohol consumption and metabolic dysfunction independently disrupt gut microbiota composition and intestinal barrier integrity, facilitating translocation of microbial-derived products to the liver via the portal circulation [61,62]. These signals activate hepatic inflammatory pathways, exacerbate insulin resistance, and promote fibrogenesis. Altered bile acid signaling and microbiota-derived metabolites further reinforce metabolic reprogramming and hepatic vulnerability.

To improve the interpretability of the evidence discussed above, the main lifestyle factors, associated clinical outcomes, mechanistic pathways, and clinical implications are summarized in Table 2.

These epidemiological associations are supported by several convergent biological mechanisms linking lifestyle exposures to metabolic and hepatic injury.

## 5. Mechanistic Pathways Linking Lifestyle Factors, Metabolic Dysfunction, and Alcohol-Related Liver Disease

Beyond their epidemiological associations, alcohol consumption and other modifiable lifestyle factors converge on a limited number of shared mechanistic pathways that link metabolic syndrome to alcohol-related liver disease. Central to this interaction is lifestyle-induced metabolic reprogramming, through which insulin resistance, chronic low-grade inflammation, oxidative stress, mitochondrial dysfunction, and gut–liver axis dysregulation act synergistically to promote hepatic injury and disease progression [8,24,31,42].

Insulin resistance represents a key initiating mechanism at the intersection of metabolic syndrome and alcohol-related liver disease. Peripheral insulin resistance increases adipose tissue lipolysis and hepatic free fatty acid influx, promoting hepatic steatosis and lipotoxicity. In the context of alcohol exposure, these metabolic alterations impair hepatic lipid oxidation and exacerbate triglyceride accumulation, thereby sensitizing hepatocytes to alcohol-induced oxidative and inflammatory injury [1,21,35]. The coexistence of metabolic dysfunction and alcohol consumption thus amplifies disturbances in hepatic glucose and lipid metabolism beyond the effects of either exposure alone [11,13,42].

Beyond metabolic and lifestyle factors, interindividual susceptibility to alcohol-related liver injury is further modulated by genetic determinants. Genetic polymorphisms affecting alcohol metabolism, inflammatory signaling, and fibrogenic pathways have been shown to influence both alcohol dependence and the severity of alcohol-related liver disease, contributing to heterogeneous disease trajectories among individuals with comparable exposure profiles [7].

Chronic low-grade inflammation further reinforces this pathogenic synergy. Metabolic syndrome is characterized by adipose tissue dysfunction and the release of pro-inflammatory cytokines, including tumor necrosis factor-α and interleukin-6, which promote hepatic insulin resistance and inflammatory signaling [8,25]. Alcohol consumption independently activates innate immune pathways within the liver, including Kupffer cell activation and inflammasome signaling [8,48]. When combined, these inflammatory inputs generate a sustained pro-inflammatory hepatic milieu that accelerates fibrogenesis and disease progression [9,12].

Oxidative stress and disruption of redox homeostasis constitute another critical mechanistic link. Alcohol metabolism induces reactive oxygen species generation through alcohol dehydrogenase, aldehyde dehydrogenase, and cytochrome P450 2E1 pathways, while metabolic dysfunction is associated with baseline oxidative stress due to impaired mitochondrial function and excess lipid substrates [8,53,54]. The convergence of these processes overwhelms hepatic antioxidant defenses, leading to lipid peroxidation, protein modification, and DNA damage, which further propagate inflammatory and fibrotic responses [9,25].

Mitochondrial dysfunction represents a downstream consequence of these metabolic and oxidative disturbances. Both metabolic syndrome and chronic alcohol exposure impair mitochondrial β-oxidation, reduce respiratory chain efficiency, and limit metabolic flexibility [8,21]. This mitochondrial vulnerability compromises hepatocellular energy homeostasis and enhances susceptibility to alcohol-related injury, reinforcing the progression from steatosis to steatohepatitis and fibrosis [11,35].

Emerging evidence also highlights the gut–liver axis as an integrative pathway linking lifestyle factors, metabolic dysfunction, and liver injury. Alcohol consumption and poor dietary patterns disrupt gut microbiota composition and increase intestinal permeability, facilitating the translocation of microbial-derived products such as lipopolysaccharides into the portal circulation [31,48]. These signals activate hepatic toll-like receptor pathways and amplify inflammatory and fibrogenic signaling. Metabolic syndrome further modifies bile acid signaling and gut microbial metabolism, reinforcing gut-derived inflammatory inputs to the liver [49,50].

Together, these convergent mechanisms provide a biological framework explaining why the coexistence of metabolic dysfunction and alcohol exposure results in accelerated liver disease progression. Rather than acting independently, modifiable lifestyle factors interact through shared metabolic and inflammatory pathways, supporting the emerging concept of metabolic alcohol-related liver disease (MetALD) as a distinct yet overlapping entity within the steatotic liver disease spectrum [13,42,43]. This mechanistic integration underscores the importance of comprehensive lifestyle interventions targeting both metabolic health and alcohol exposure to mitigate liver disease risk and progression [28,29].

## 6. Discussion and Clinical Implications

**Figure 1:**
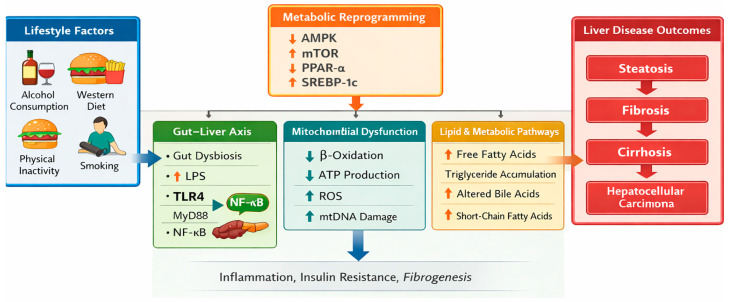
Mechanistic framework linking lifestyle factors to metabolic reprogramming and liver disease progression. Lifestyle exposures including alcohol consumption, Western dietary patterns, smoking, and physical inactivity promote metabolic reprogramming through dysregulation of key metabolic pathways such as AMPK, mTOR, and PPAR-α. These alterations interact with gut–liver axis signaling (LPS–TLR4–NF-κB, NLRP3 inflammasome activation), mitochondrial dysfunction (impaired β-oxidation, reduced ATP production, and increased reactive oxygen species generation), and altered lipid metabolism. Together, these processes contribute to hepatic inflammation, fibrogenesis, and progression toward cirrhosis and hepatocellular carcinoma. Abbreviations: AMPK, AMP-activated protein kinase; mTOR, mechanistic target of rapamycin; PPAR-α, peroxisome proliferator-activated receptor alpha; LPS, lipopolysaccharide; TLR4, Toll-like receptor 4; NF-κB, nuclear factor kappa B; NLRP3, NOD-like receptor pyrin domain-containing protein 3; ATP, adenosine triphosphate.

This narrative review highlights lifestyle-induced metabolic reprogramming as a central pathogenic mechanism linking metabolic syndrome and alcohol-related liver disease, operating through coordinated alterations in metabolic pathways governing insulin sensitivity, lipid handling, mitochondrial function, and inflammatory signaling. The findings consistently demonstrate that alcohol consumption, smoking, unhealthy dietary patterns, and physical inactivity converge on insulin resistance, lipid dysregulation, chronic low-grade inflammation, and mitochondrial dysfunction, thereby amplifying both systemic metabolic risk and hepatic injury [6,7,8,9,10,11,12,25,44]. Collectively, these lifestyle exposures reshape metabolic homeostasis and increase liver-specific vulnerability to injury, as illustrated in Figure 1.

Alcohol-related liver disease remains a major cause of chronic liver morbidity and mortality worldwide, with evolving epidemiological patterns shaped by lifestyle behaviors, metabolic risk factors, and socioeconomic context [55].

Although primary studies focusing on MASLD/MASH were excluded to avoid conflation with alcohol-related liver disease, contemporary MASLD-based classification frameworks were referenced to ensure terminological consistency and alignment with current disease taxonomy. Importantly, these sources were used solely for conceptual framing, while all analytical conclusions regarding disease associations and outcomes were derived from ALD-focused evidence.

In this broader context, metabolic dysfunction has emerged as a shared driver of hepatocellular carcinoma risk across steatotic liver disease phenotypes, reinforcing the oncogenic relevance of metabolic reprogramming even beyond alcohol-related etiologies [56].

Alcohol consumption emerged as the dominant lifestyle factor influencing both metabolic and hepatic outcomes. Beyond its direct hepatotoxic effects, alcohol exerts profound systemic metabolic consequences, including impaired insulin signaling, altered lipid handling, and activation of inflammatory pathways [4,8,9,44]. Cohort and synthesis level evidence supports dose and pattern dependent effects of alcohol on MetS risk and ALD progression, with binge drinking conferring additional risk independent of average intake [30,36,37,38]. Importantly, metabolic abnormalities appear to lower the threshold for alcohol-induced liver injury, resulting in accelerated fibrosis progression and increased risk of cirrhosis and hepatocellular carcinoma (HCC) in metabolically vulnerable individuals [6,7,10,11,39,40,41].

At the population level, alcohol consumption contributes substantially to premature mortality and years of life lost, with marked regional variability and a disproportionate burden observed in Eastern European populations [58,59].

A key finding across the reviewed literature is the synergistic interaction between metabolic dysfunction and alcohol exposure in determining liver disease severity. The coexistence of MetS components such as obesity, insulin resistance, and dyslipidemia with alcohol consumption amplifies oxidative stress, promotes hepatic lipotoxicity, and exacerbates inflammatory and fibrogenic responses [7,8,25]. This dual hit paradigm has been increasingly conceptualized as metabolic alcohol-related liver disease (MetALD), a framework that integrates metabolic and alcohol-related etiologies into a unified disease continuum [13,42,43]. Recognition of MetALD has important clinical implications, as it underscores the need for comprehensive metabolic risk assessment in individuals with alcohol exposure and challenges traditional dichotomous classifications of liver disease etiology.

Importantly, the combined presence of metabolic dysfunction and alcohol consumption has also been associated with increased all-cause and liver-related mortality, highlighting their synergistic prognostic impact [59].

Smoking represents an additional modifiable factor that adversely affects both metabolic and hepatic health. Evidence from large cohort studies indicates a dose-dependent association between cigarette smoking and MetS, mediated in part through increased insulin resistance and systemic inflammation [14,15,16,17]. In the context of ALD, smoking appears to act as a disease modifier, exacerbating liver injury through oxidative stress, inflammatory signaling, and potential effects on gut liver axis integrity [8,9]. These findings support the inclusion of smoking cessation as a core component of lifestyle-based interventions targeting metabolic and liver disease risk.

Conversely, physical activity consistently emerged as a protective factor against metabolic dysfunction and liver disease progression. Regular moderate to vigorous physical activity was associated with improved insulin sensitivity, favorable lipid profiles, and reduced MetS prevalence across diverse populations [19,20]. Importantly, cohort data suggest that physical activity may attenuate ALD risk even among individuals with harmful alcohol use, highlighting its potential to partially counterbalance alcohol related metabolic and hepatic injury [18,26]. Mechanistically, physical activity enhances mitochondrial function, reduces hepatic fat accumulation, and modulates inflammatory pathways, reinforcing its central role in metabolic homeostasis.

These benefits are particularly relevant in fatty liver disease, where exercise-induced improvements in insulin sensitivity may mitigate metabolic–alcohol-related hepatic injury [61].

Dietary patterns also play a critical role in shaping metabolic and hepatic outcomes. Western dietary patterns, characterized by high intakes of saturated fats and refined carbohydrates, were consistently associated with metabolic deterioration and increased MetS risk [21]. In contrast, adherence to Mediterranean-style dietary patterns was linked to improved metabolic profiles and reduced cardiometabolic risk [46]. Emerging evidence further suggests that diet quality interacts with alcohol exposure to influence liver disease trajectories, with poor nutritional status exacerbating alcoholic fatty liver and fibrosis progression [9,22,23,47]. These observations underscore the metabolic relevance of diet in ALD and support integrated nutritional strategies in disease prevention and management.

Accordingly, nutritional support has been recognized as a core therapeutic component in alcohol-related liver disease, contributing to improved metabolic control and clinical outcomes [62].

From a clinical and public health perspective, the findings of this review emphasize that MetS and ALD should not be viewed as isolated entities but rather as interconnected manifestations of lifestyle-driven metabolic dysregulation. Integrated lifestyle-based interventions targeting alcohol consumption, smoking cessation, diet quality, and physical activity are therefore likely to yield synergistic benefits by addressing shared metabolic and inflammatory pathways [27,28,29]. Such approaches align with recent guideline recommendations and position statements advocating for holistic risk factor modification in chronic liver disease [42,43].

The cumulative burden of adverse lifestyle factors has been shown to markedly increase metabolic risk, supporting comprehensive lifestyle-based prevention strategies [63,64].

Moreover, effective treatment of alcohol use disorder represents a key determinant of prognosis in patients with advanced liver disease, with sustained abstinence associated with improved survival, and with regression of fibrosis markers in patients with metabolic dysfunction [65].

Recent multi-omics approaches have further highlighted the role of metabolic reprogramming in hepatocellular carcinoma (HCC) development and progression. Integrated transcriptomic, metabolomic, and experimental analyses indicate that dysregulation of inflammatory and metabolic signaling pathways—including NF-κB-mediated inflammation, oxidative stress responses, and alterations in cellular energy metabolism—contributes to hepatocarcinogenesis by promoting tumor cell proliferation, survival, and fibrogenic microenvironment remodeling. These findings support the concept that metabolic disturbances associated with chronic liver disease can facilitate oncogenic transformation through coordinated changes in metabolic and inflammatory networks, reinforcing the importance of metabolic pathway modulation in preventing liver cancer progression [66,67].

Taken together, the available evidence supports a hierarchical and interactive model of lifestyle-related risk in metabolic and alcohol-related liver disease. Alcohol consumption emerges as the dominant and most directly hepatotoxic factor, while metabolic dysfunction acts as a critical susceptibility modifier that amplifies liver injury. In contrast, smoking appears to function primarily as a disease modifier, and dietary patterns and physical activity exert broader metabolic effects that indirectly influence hepatic outcomes. Importantly, these exposures do not act in isolation but converge through shared pathways of metabolic reprogramming, inflammation, and mitochondrial dysfunction. This integrated perspective reinforces the concept of metabolic alcohol-related liver disease (MetALD) as a systems-level condition driven by clustered lifestyle factors rather than single exposures.

### 6.1. Clinical and Public Health Implications

Recognition of lifestyle-driven metabolic reprogramming as a unifying mechanism linking MetS and ALD has important clinical and public health implications. Integrated risk assessment strategies should include systematic evaluation of metabolic risk factors in individuals with harmful alcohol use, as well as careful assessment of alcohol consumption patterns in patients with metabolic syndrome.

Lifestyle-based interventions—including alcohol reduction or abstinence, smoking cessation, dietary optimization, and promotion of regular physical activity—represent the cornerstone of management within the MetALD framework. Integrated prevention strategies targeting clustered lifestyle behaviors are likely to yield synergistic benefits at both individual and population levels.

### 6.2. Limitations and Future Directions

As a narrative review, this synthesis does not provide quantitative effect estimates and is subject to limitations inherent in the underlying observational literature, including residual confounding and heterogeneity in exposure assessment. Nevertheless, the consistency of findings across diverse populations and study designs supports the biological plausibility and clinical relevance of the MetALD concept.

Future research should prioritize longitudinal and interventional studies to refine MetALD-specific risk stratification, elucidate causal metabolic pathways, and evaluate the long-term impact of integrated lifestyle interventions on liver-related outcomes.

Based on the evidence synthesized in this review, several testable research directions can be proposed to guide future investigations into metabolic alcohol-related liver disease. The coexistence of metabolic dysfunction and harmful alcohol consumption may accelerate liver disease progression through amplification of metabolic reprogramming pathways involving insulin resistance, mitochondrial dysfunction, oxidative stress, and inflammatory signaling. Integrated lifestyle interventions combining alcohol reduction or abstinence, dietary optimization, and structured physical activity may lead to greater improvements in hepatic metabolic profiles and fibrosis risk compared with single-factor interventions in individuals with metabolic alcohol-related liver disease (MetALD). Alterations in gut microbiota composition and gut-derived metabolites, including bile acid derivatives and short-chain fatty acids, may act as mediators linking lifestyle exposures to hepatic metabolic reprogramming and could represent potential biomarkers of MetALD progression. In addition, distinct metabolomic signatures reflecting disturbances in lipid metabolism, tricarboxylic acid cycle intermediates, and oxidative stress pathways may help identify early stages of metabolic–alcohol-related liver injury before clinically detectable fibrosis develops. Finally, sex-related differences in alcohol metabolism, hormonal regulation, adipose tissue distribution, and inflammatory signaling may influence susceptibility to metabolic reprogramming and contribute to differential progression of metabolic alcohol-related liver disease between men and women.

### 6.3. Clinical Takeaways for Practice

Based on the evidence synthesized in this review, several practical clinical implications can be highlighted. Alcohol reduction or sustained abstinence remains the most effective intervention for preventing the progression of alcohol-related liver disease and improving survival, particularly in advanced disease stages. Consequently, systematic assessment of alcohol consumption should be incorporated into the clinical evaluation of patients with metabolic dysfunction.

Bidirectional clinical screening appears warranted. Individuals with harmful alcohol use should be evaluated for components of metabolic syndrome, while patients presenting with metabolic dysfunction should be systematically assessed for alcohol consumption patterns. Recognizing this overlap is essential for identifying patients at risk within the emerging MetALD framework.

Integrated lifestyle interventions targeting clustered behavioral risk factors—including alcohol consumption, smoking, dietary quality, and physical activity—are likely to produce synergistic metabolic and hepatic benefits. Regular physical activity should be encouraged, as it improves insulin sensitivity, mitochondrial function, and hepatic lipid metabolism. In parallel, nutritional optimization and adherence to high-quality dietary patterns, such as Mediterranean-style diets, may improve metabolic profiles and potentially mitigate liver disease progression.

Smoking cessation should also be considered an integral component of liver disease management, given its association with metabolic dysfunction, oxidative stress, and fibrosis progression. Overall, comprehensive clinical risk stratification should incorporate both metabolic and alcohol-related risk factors, reflecting the growing recognition of their interaction within the MetALD spectrum.

### 6.4. Research Priorities

Based on the current evidence gaps, several priority research directions can be identified. Future prospective longitudinal studies are needed to better quantify the interaction between metabolic dysfunction and alcohol exposure in determining liver disease progression and clinical outcomes. Randomized lifestyle intervention trials should evaluate the combined impact of alcohol reduction, dietary modification, and structured physical activity on metabolic and hepatic outcomes in populations with metabolic alcohol-related liver disease.

Mechanistic investigations integrating metabolomics and microbiome profiling may further clarify the biological pathways linking lifestyle-induced metabolic reprogramming to liver injury. In addition, sex-disaggregated analyses are necessary to better understand biological differences in susceptibility to metabolic and alcohol-related liver disease.

Finally, the development of risk stratification tools that integrate both metabolic and alcohol-related exposures could improve early identification of high-risk MetALD phenotypes and facilitate more personalized prevention and management strategies.

## 7. Conclusions

Metabolic syndrome and alcohol-related liver disease represent interconnected manifestations of lifestyle-driven metabolic dysregulation rather than isolated clinical entities. Evidence synthesized in this review indicates that modifiable lifestyle factors—including alcohol consumption, smoking, unhealthy dietary patterns, and physical inactivity—converge on shared metabolic and inflammatory pathways that promote insulin resistance, mitochondrial dysfunction, oxidative stress, and chronic low-grade inflammation.

The concept of metabolic alcohol-related liver disease (MetALD) provides a useful framework for understanding the synergistic interaction between metabolic dysfunction and alcohol exposure in accelerating liver disease progression. Recognition of this dual etiology highlights the need for integrated clinical approaches that simultaneously address metabolic risk factors and alcohol-related harm.

From a clinical perspective, early identification of individuals with combined metabolic and alcohol-related risk profiles is essential for effective prevention and management. Comprehensive lifestyle-based strategies—including alcohol reduction or abstinence, smoking cessation, dietary optimization, and promotion of regular physical activity—may provide synergistic benefits by targeting the shared metabolic pathways underlying disease progression.

Future research integrating metabolomics, microbiome profiling, and longitudinal clinical studies will be critical for improving risk stratification and developing targeted interventions within the emerging MetALD framework.

## Figures and Tables

**Table 1:** Comparative overview of metabolic syndrome, alcohol-related liver disease, MASLD, and metabolic alcohol-related liver disease.

Feature	Metabolic Syndrome (MetS)	Alcohol-Related Liver Disease (ALD)	MASLD	MetALD
Primary definition	Cluster of cardiometabolic risk factors including central obesity, insulin resistance, dyslipidemia, and hypertension	Liver injury primarily caused by harmful alcohol consumption	Steatotic liver disease associated with metabolic dysfunction	Liver disease characterized by coexistence of metabolic dysfunction and clinically relevant alcohol consumption
Main etiological drivers	Obesity, insulin resistance, sedentary lifestyle, poor diet	Chronic excessive alcohol intake	Metabolic dysfunction (obesity, insulin resistance, type 2 diabetes)	Combined metabolic dysfunction and alcohol exposure
Principal affected organ/system	Systemic metabolic disorder affecting multiple organs	Liver	Liver	Liver
Key pathophysiological mechanisms	Insulin resistance, dysregulated lipid metabolism, chronic low-grade inflammation	Oxidative stress, acetaldehyde toxicity, mitochondrial dysfunction, inflammation	Hepatic steatosis, lipotoxicity, insulin resistance, inflammation	Synergistic metabolic dysfunction and alcohol-induced hepatotoxicity
Typical clinical manifestations	Central obesity, hypertension, hyperglycemia, dyslipidemia	Hepatic steatosis, alcoholic hepatitis, fibrosis, cirrhosis	Hepatic steatosis ± steatohepatitis (MASH), fibrosis	Accelerated fibrosis progression, cirrhosis, hepatocellular carcinoma risk
Relationship with metabolic dysfunction	Core defining component	Frequently aggravated by metabolic risk factors	Central defining mechanism	Combination of metabolic dysfunction and alcohol exposure
Relationship with alcohol consumption	Not required	Primary etiological factor	Minimal or absent alcohol exposure	Moderate or significant alcohol exposure combined with metabolic dysfunction
Clinical relevance	Major risk factor for cardiovascular disease and metabolic liver disease	Leading cause of chronic liver disease worldwide	Most common cause of steatotic liver disease	Emerging phenotype integrating metabolic and alcohol-related liver injury

**Table 2:** Summary of modifiable lifestyle factors influencing metabolic syndrome and alcohol-related liver disease outcomes.

Lifestyle Factor	Main Clinical Outcomes	Consistency of Evidence	Key Mechanistic Pathways	Clinical Implications
Alcohol consumption	Fibrosis progression, cirrhosis, hepatocellular carcinoma (HCC), liver-related mortality	Strong and consistent across cohort studies and meta-analyses	Oxidative stress, mitochondrial dysfunction, impaired lipid metabolism, inflammatory signaling	Alcohol reduction or abstinence is the most effective strategy to prevent disease progression
Smoking	Increased risk of metabolic syndrome, accelerated fibrosis progression, worse liver disease severity	Moderate but consistent observational evidence	Oxidative stress, inflammatory activation, hepatic stellate cell activation	Smoking cessation should be included in integrated lifestyle interventions
Dietary patterns	Increased risk of metabolic syndrome and steatosis progression with Western diets; improved metabolic profiles with Mediterranean diet	Moderate evidence, predominantly observational and synthesis-level studies	Lipotoxicity, insulin resistance, altered gut–liver axis signaling	Promotion of high-quality dietary patterns (e.g., Mediterranean diet)
Physical activity	Reduced metabolic syndrome risk, lower fibrosis risk, improved metabolic profiles	Moderate to strong observational evidence	Improved insulin sensitivity, enhanced mitochondrial function, reduced hepatic fat accumulation	Regular moderate–vigorous physical activity recommended for metabolic and liver health

## Data Availability

No new data were created or analyzed in this study. Data sharing is not applicable to this article.

## Abbreviations

ALD	alcohol-related liver disease
ArLD	alcohol-associated liver disease
AMPK	AMP-activated protein kinase
ATP	adenosine triphosphate
CD14	cluster of differentiation 14
CYP2E1	cytochrome P450 2E1
FFA	free fatty acids
HCC	hepatocellular carcinoma
HSC	hepatic stellate cell
IKKε	IκB kinase epsilon
IRAK	interleukin-1 receptor-associated kinase
IRF3	interferon regulatory factor 3
LBP	lipopolysaccharide-binding protein
LPS	lipopolysaccharide
MAPK	mitogen-activated protein kinase
MASLD	metabolic dysfunction–associated steatotic liver disease
MASH	metabolic dysfunction–associated steatohepatitis
MD-2	myeloid differentiation protein 2
MetALD	metabolic alcohol-related liver disease
MetS	metabolic syndrome
mtDNA	mitochondrial DNA
mTOR	mechanistic target of rapamycin
NF-κB	nuclear factor kappa B
NLRP3	NOD-like receptor pyrin domain-containing protein 3
PPAR	peroxisome proliferator-activated receptor
PPAR-α	peroxisome proliferator-activated receptor alpha
ROS	reactive oxygen species
TBK1	TANK-binding kinase 1
TCA cycle	tricarboxylic acid cycle
TGF-β	transforming growth factor beta
TLR	Toll-like receptor
TLR4	Toll-like receptor 4
TRAF6	tumor necrosis factor receptor-associated factor 6
TRIF	TIR-domain-containing adapter-inducing interferon-β
